# T Follicular Helper Cells As a New Target for Immunosuppressive Therapies

**DOI:** 10.3389/fimmu.2017.01510

**Published:** 2017-11-07

**Authors:** Lin Yan, Kitty de Leur, Rudi W. Hendriks, Luc J. W. van der Laan, Yunying Shi, Lanlan Wang, Carla C. Baan

**Affiliations:** ^1^Department of Laboratory Medicine, West China Hospital, Sichuan University, Chengdu, China; ^2^Department of Internal Medicine, Division of Nephrology and Transplantation, Erasmus MC, University Medical Center, Rotterdam, Netherlands; ^3^Department of Surgery, Erasmus MC, University Medical Center, Rotterdam, Netherlands; ^4^Department of Pulmonary Medicine, Erasmus MC, University Medical Center, Rotterdam, Netherlands; ^5^Department of Nephrology, West China Hospital, Sichuan University, Chengdu, China

**Keywords:** T follicular helper cells, immunosuppression, humoral immunity, transplantation, allograft rejection, donor-specific antibodies, costimulation, cytokines

## Abstract

Over the past decade, antibody-mediated (humoral) rejection has been recognized as a common cause of graft dysfunction after organ transplantation and an important determinant for graft loss. In humoral alloimmunity, T follicular helper (Tfh) cells play a crucial role, because they help naïve B cells to differentiate into memory B cells and alloantibody-producing plasma cells within germinal centers. In this way, they contribute to the induction of donor-specific antibodies, which are responsible for the humoral immune response to the allograft. In this article, we provide an overview of the current knowledge on the effects of immunosuppressive therapies on Tfh cell development and function, and discuss possible new approaches to influence the activity of Tfh cells. In addition, we discuss the potential use of Tfh cells as a pharmacodynamic biomarker to improve alloimmune-risk stratification and tailoring of immunosuppression to individualize therapy after transplantation.

## Introduction

Organ transplantation is the treatment of choice for end-stage organ failure. Although current immunosuppressive regimens are effective in the short-term, long-term allograft survival rates are still suboptimal with rejection being the leading cause of graft loss (1). Allograft rejection can develop from either cellular or humoral immune responses against the allograft, or from “mixed rejection” involving both types of responses (2). In particular, humoral anti-donor reactivity *via* the formation of donor-specific antibodies (DSA) is associated with poor allograft outcomes (3–5). Formation of DSA relies on antigen-activated T follicular helper (Tfh) cells, which are located in the germinal centers (GC) where they provide help to antigen-activated B cells, which in turn respond by differentiating into immunoglobulin-producing plasma cells and high-affinity memory B cells (6, 7).

B cell depleting therapies have been used to control the formation of DSA in transplant recipients (8) but are not generally used as maintenance treatment because of the risk of side effects. Based on their pivotal role in regulating humoral immunity it can be postulated that Tfh cells, rather than B cells, could be targeted to inhibit the development of antibody-mediated anti-donor reactivity. Currently, no Tfh-specific agents have been evaluated in phase II or III trials. Several animal studies and a small number of clinical studies in organ transplant recipients have demonstrated the importance of Tfh cells in the process of alloantibody production (9). The specific effects of immunosuppressive therapies on Tfh cell activity, however, are less established and now subject to many ongoing research efforts. In this article, we summarize current knowledge on the interplay between immunosuppressive drugs and the generation and function of Tfh cells, and consider new biological targets that might influence the proliferation, differentiation, and activity of Tfh cells.

## Biology of Tfh Cells

### Differentiation of Tfh Cells

**Figure 1:**
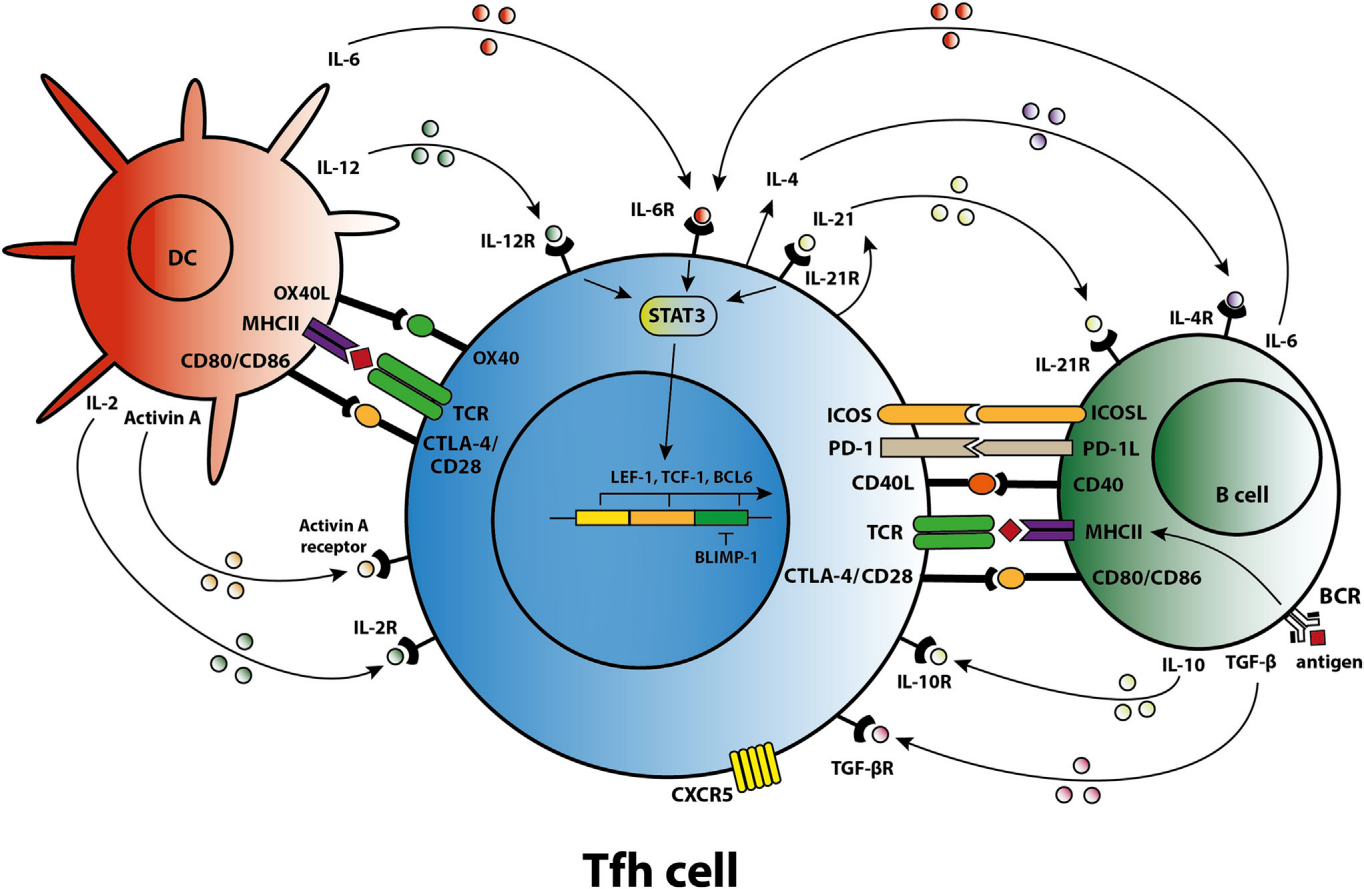
T follicular helper (Tfh) cell differentiation, activation, and crosstalk. Schematic overview of molecules involved in the differentiation of Tfh cells, the activation of Tfh cells by dendritic cells (DCs) and B cells, and the crosstalk of Tfh cells with DCs and B cells.

Differentiation of a human naïve CD4^+^ T cell into a Tfh cell is a complex and dynamic process involving multiple stages (10). A combination of signals determines whether the naïve T cell differentiates toward a Th1, Th2, Th17, or Tfh subset including the expression of specific transcription factors, signal transducer and activator of transcription (STAT) proteins, cytokines, and chemokine receptors that allow the T cell to migrate to the site of inflammation. When a naïve T cell expresses C–C chemokine receptor 7 (CCR7), migration is promoted to T cell-rich zones in secondary lymphoid organs (SLO) and tertiary lymphoid structures present in chronically inflamed organs. Protein activin A [a member of the transforming growth factor-β (TGF-β) superfamily] is present locally after the T cell encounters an antigen-presenting dendritic cell (DC) and mediates downregulation of CCR7, followed by upregulation of C–X–C chemokine receptor 5 (CXCR5) (11). Expression of CXCR5 is essential for localization of the Tfh cells at the T–B border of B-cell-rich follicles, where Tfh cells interact with B cells that recognize antigen *via* their B-cell receptor (BCR) (Figure 1). Sequential antigen presentation by DCs and B cells is required for optimal differentiation of Tfh cells and the subsequent GC reaction (12). After cognate antigen recognition, Tfh cells migrate inside the B-cell follicles and develop into activated GC–Tfh cells, which orchestrate the development of high-affinity GC B cells. In addition to CXCR5, activated Tfh cells express the coinhibitory protein programmed death 1 (PD-1) and inducible T-cell costimulatory molecule (ICOS) (7, 9). Recently, it has been demonstrated in a conditional knock out mouse model that Tfh cells express the transcription factors lymphoid enhancer binding factor 1 and T cell factor 1, both of which are involved in regulation of the Tfh transcriptional repressor B cell lymphoma 6 (Bcl-6) (13). These transcription factors promote early Tfh cell differentiation by sustaining the expression of IL-6Rα and gp130, and by promoting upregulation of ICOS and expression of Bcl-6 which is also known as the master transcription factor for Tfh cells and represses transcription of among others *B lymphocyte-induced maturation protein-1* (*Blimp-1*), *T-box transcription factor* (*T-bet*) (Th1 development), and *RAR-related orphan receptor γt* (RORγ*t*) (Th17 development) (14, 15). Apart from being present in SLOs, CXCR5^+^CD4^+^ Tfh cells are also present in blood, representing approximately 10% of human circulating memory CD4^+^ T cells (16, 17). Memory Tfh cells form a heterogeneous population based on the expression of the chemokine receptors CXCR3 and CCR6: CXCR3^−^CCR6^−^ represent Tfh2 cells, CXCR3^+^CCR6^−^ represent Tfh1 cells, and CXCR3^−^CCR6^+^ represent Tfh17 cells (16). These subsets have distinct capacities to help B cells, the CXCR3^−^ Tfh2 and Tfh17 cells promote B cell differentiation toward immunoglobulin-producing cells *via* secretion of IL-21, whereas CXCR3^+^ Tfh1 cells lack this function (18, 19). In addition, the Tfh2 cells promote particularly IgG and IgE secretion, whereas Tfh17 cells are more efficient in promoting IgG and IgA secretion (16). Overall, an appropriate microenvironment is essential for coordination of Tfh cell lineage differentiation.

### Cytokines Involved in Tfh Cell Differentiation, Activation, and Function

Coordinated activity by cytokines triggers specific transcription programs that stimulate the expression of molecules responsible for the effector function of Tfh cells (7). The differentiation of naïve human CD4^+^ T cells in the SLOs toward a Tfh cell phenotype is primarily mediated by IL-12, IL-6, and TGF-β signaling. Activin A, in combination with IL-12, mediates an early shift toward the Tfh phenotype, including skewing toward expression of IL-21 (11, 20). IL-12 production is profoundly increased by activated DCs in the T cell-rich zone (21). TGF-β is another cytokine involved in human Tfh cell differentiation that after binding to its receptor, phosphorylates the transcription factors STAT3 and STAT4, key steps in the Tfh cell differentiation process (22). As well as IL-12 and TGF-β, IL-6 contributes to differentiation into Tfh cells. One clinical study, for example, showed that secretion of IL-6 by plasmablasts resulted in Tfh cell differentiation (23). Of note, interplay between IL-6 and IL-21 is required to achieve optimal Tfh cell differentiation and function, although an absence of either IL-6 or IL-21 in a mouse model does not fully abolish Tfh cell formation (24, 25). By contrast, GC formation and the differentiation of B cells into immunoglobulin-producing plasmablasts are dependent on IL-21 producing Tfh cells (25, 26).

### Inhibition of Tfh Cells

T follicular helper cell function depends on the balance between pro-inflammatory and anti-inflammatory signals. Several factors have been reported to control Tfh cell activation. Recently, it became evident that a subset of regulatory T (Treg) cells—the follicular regulatory T (Tfr) cells—express Foxp3, Bcl-6, and CXCR5 and have the capacity to regulate the Tfh-driven GC reaction (27–29). However, the immunoregulatory mechanisms by which Tfr cell functions are controlled are largely unknown. Animal studies have shown that the coinhibitory receptor cytotoxic T-lymphocyte-associated protein 4 (CTLA4) that is highly expressed by Tfr cells and moderately expressed on Tfh cells is involved in the suppressive effects of these cells. Tfr cells lacking the coinhibitory molecule CTLA4 have an impaired ability to inhibit B cell antibody production (30, 31). Conversely, mice deficient for PD-1 on Tfr cells have increased suppressive activity, since PD-1 controls the activation of Tfr cells (32). In kidney transplant patients who received anti-CD20 rituximab induction therapy, both Tfh and Tfr cells remained in the lymph nodes despite disruption of the GC and elimination of B cells (33), underlining their independent mechanism of action. The role of Tfr cells in preventing rejection of the allograft is largely unknown. Recently, Chen *et al*. showed that the ratio of Tfr cells in peripheral blood and renal graft biopsies from patients with antibody-mediated rejection (AMR) was significantly lower than in non-AMR patients, whereas Tfh2 and Tfh17 ratios increased, suggesting that increased Tfh activation levels contribute to AMR (34).

Anti-inflammatory cytokines secreted by Tfr cells also influence Tfr cell function. The pleiotropic cytokine IL-10 inhibits antibody production *via* regulation of the quantity and quality of Tfh cells in mice immunized with sheep red blood cells (35). By contrast, lymphocytic choriomeningitis virus (LCMV)-infected mice deficient for IL-10 had lower frequencies of virus-specific Tfh cells but no decrease in GC B cells or LCMV-specific antibodies (36). IL-2 is a critical factor for the regulation of Tfh–B cell interaction *in vivo*. Although originally recognized as an essential T-cell growth factor, IL-2 suppresses Tfh cell differentiation *via* activation of Blimp-1 resulting in a hampered formation of antigen-specific B cells and IgG responses in mice infected with the influenza virus (37, 38). Ray *et al*. recently demonstrated that IL-2-mediated activation of Akt kinase and mTORc1 was necessary to shift differentiation to Th1, with less differentiation to Tfh cells (39). By contrast, IL-21 inhibits the expression of CD25 (part of the IL-2 receptor) in a Bcl-6-dependent manner (40).

### Tfh–B Cell Crosstalk

Cytokines and costimulatory molecules secreted by B cells are able to encourage activation of Tfh cells within the GC (Figure 1). Moreover, apart from their role as an antigen-presenting cell (APC), B cells contribute to the activation and regulation of the Tfh cells *via* secretion of cytokines like IL-6 and IL-10 (41, 42). Meanwhile, Tfh cells are involved in the GC reaction to promote B-cell activation. The GC consists of a polarized structure with two compartments that are designed for proliferation and affinity selection. Within the dark zone (DZ), B cells undergo several rounds of proliferation and somatic hypermutation (SHM) in the V-region of their BCR (43). The point mutations that are created during SHM allow affinity maturation and lead to antibody diversity. Afterward, the DZ B cells migrates to the light zone (LZ), where they capture antigen presented by follicular DC networks and present it on MHC class II molecules to cognate Tfh cells (44). The amount of antigen captured by the B cell and presented to Tfh cells in the LZ directly corresponds to the amount of B-cell division and hypermutation in the DZ (45). Thus, T-cell help and not direct competition for antigen is the limiting factor in GC selection (46). High-affinity B cells present antigen to cognate Tfh cells, triggering a signaling pathway that allows (i) B-cell differentiation into long-living plasma cells, (ii) differentiation of long-lived memory B cells, or (iii) recirculation of B cells to the DZ for a new round of division and SHM (47). Activated Tfh cells produce IL-21 and IL-4, two cytokines that support B cell differentiation. Within the GC, Tfh cell function changes from IL-21^+^ Tfh cells, responsible for the selection of high-affinity B cell clones, toward IL-4^+^ Tfh cells that have high expression of CD40L and which direct B-cell class switch recombination and differentiation toward antibody-producing plasma cells (48, 49). PD-1^hi^ Tfh cells are involved in this GC reaction while PD-1^lo^ Tfh cells represent precursor memory T cells with a Tfh-like phenotype (50). In a previous study, we found co-localization of T and B cells in cellular infiltrates of renal rejection biopsies, supporting a role for T–B cell interaction within the kidney allograft (51).

### Genetic Defects Influencing Human Tfh Cell Differentiation

Several heritable monogenic defects are known to affect the function and differentiation of Tfh cells. PBMCs of patients with primary immunodeficiencies (PIDs) have been characterized in various studies (52–57). In these studies, loss-of-function (LOF) mutations in the genes encoding STAT3, ICOS, Bruton’s tyrosine kinase (BTK), CD40L, NF-κB essential modulator, and IL10R reduced the numbers of Tfh cells (52, 55, 56). LOF mutations in the genes encoding STAT3, IL21-R, and gain-of-function (GOF) mutation in the gene encoding STAT1 resulted in a phenotype with elevated levels of IFNγ and PD-1, both of which control Tfh-mediated B cell differentiation. By contrast, LOF mutations in *IFNGR1/2, STAT1*, and *IL12RB1* genes caused impaired function of IFNγ and thus promoted Tfh–B-cell interaction (52). Another study by Ma *et al*. reported that mutations in the genes encoding STAT3, IL-21R, CD40L, IFNGR1 LOF, or STAT1 GOF inhibit the differentiation of Tfh cells *via* impairing the generation of IL-21 (53). Lower frequencies of Tfh cells were observed in patients with two transmembrane activator and CAML interactor mutations compared with patients with a single mutation or without mutations (57). A study by Alroqi *et al*. described the presence of Tfh cells in patients with LPS-responsive beige-like anchor (LRBA) deficiencies. LRBA promotes the intracellular transport of CTLA4 toward the cell membrane, mostly expressed on Treg cells and Th17 cells (58). In all patients studied, increased frequencies of Tfh cells were measured associated with low CTLA4 expression levels on the Treg cells (54). When these patients were treated with CTLA4-Ig therapy, the frequencies of Tfh levels significantly decreased (54). To this end, Tfh-cell frequencies may be a useful readout in patients with LRBA and CTLA4 deficiencies to monitor the effect of CTLA4-Ig therapy. Taken together, the defects in differentiation of Tfh cells found in various PID patients provide strong evidence that the genes mutated in these diseases are essential for Tfh differentiation. Moreover, the finding of reduced Tfh cells in X-linked agammaglobulinemia patients with mutations in BTK shows that Tfh development is B cell dependent (52).

## The Effects of Conventional Immunosuppression on Tfh Cells

Maintenance immunosuppression after solid organ transplantation typically consists of a calcineurin inhibitor (CNI), either tacrolimus (Tac) or cyclosporin A, the T-cell proliferation inhibitor mycophenolate mofetil and steroids. An *in vitro* study analyzing the effect of methylprednisolone and CNI agents on T cells showed that these immunosuppressants could inhibit differentiation of human naïve CD4^+^ T cells into Tfh cells (59). *In vivo*, we found that Tfh-like cells are present in the circulation of kidney transplant recipients receiving Tac-based immunosuppression. These circulating Tfh-like cells have the capacity to induce B-cell differentiation into immunoglobulin-producing plasmablasts (51). A recent study from our group demonstrated that Tac had a small inhibitory effect on Tfh-cell generation *in vitro* and could partially prevent Tfh-cell activation. The production of IL-21 was incompletely inhibited by high concentrations of Tac, which may be why the remaining activated Tfh cells retained the potential capacity to assist B cells (60). In another study, methylprednisolone treatment of patients with systemic lupus erythematosus was found to reduce the number of peripheral CD4^+^CXCR5^+^PD-1^+^ T cells, but without any evidence of altered Tfh function (61). Based on the available data, it can be concluded that conventional immunosuppressive therapies do not block Tfh-cell activity completely and Tfh-cell activity could usefully be examined in patients with allograft rejection or rapidly declining graft function.

**Figure 2:**
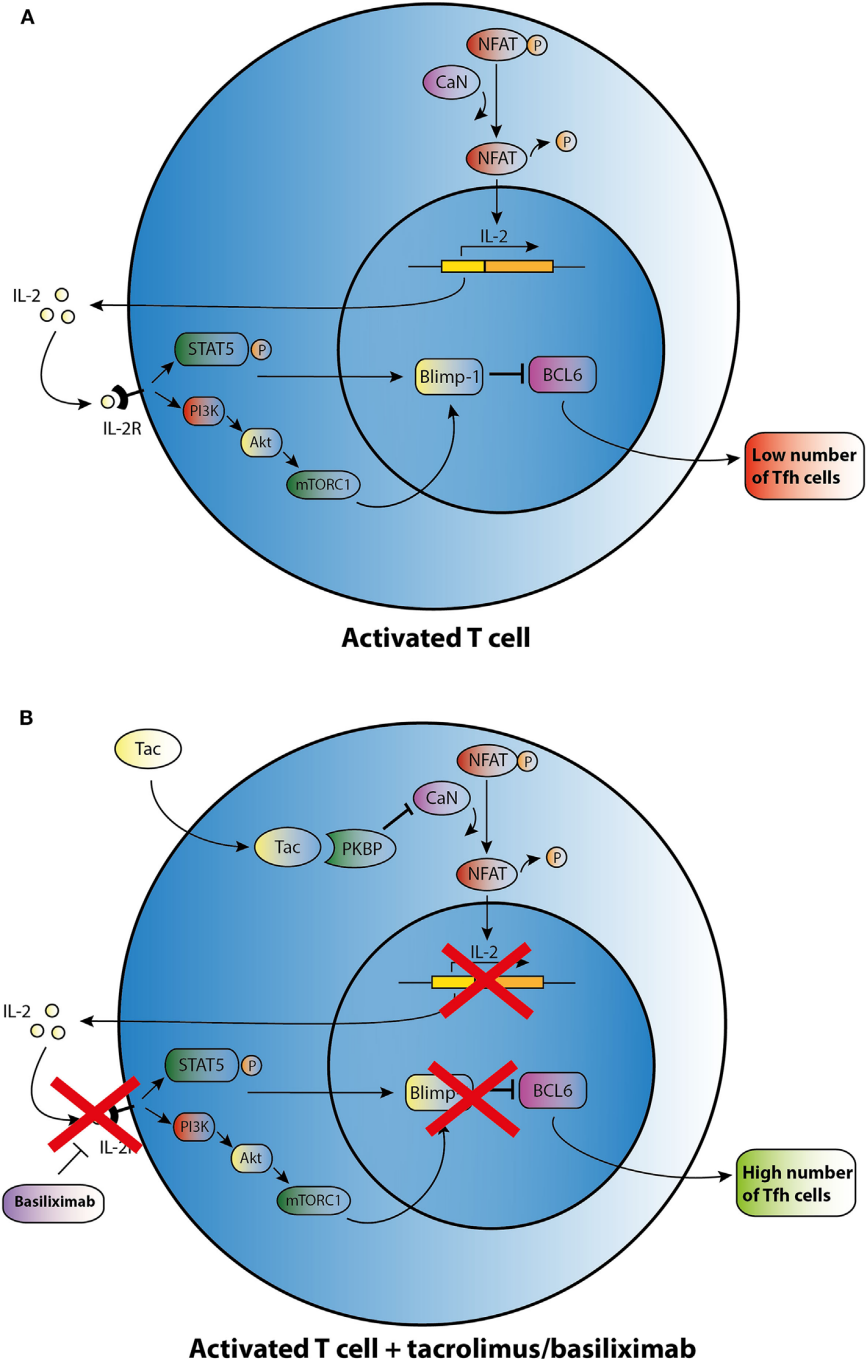
Possible effect of tacrolimus (Tac) and basiliximab on T follicular helper (Tfh) cell differentiation and activation. **(A)** An activated T cell is depicted in the upper panel. **(B)** After addition of Tac, calcineurin (CaN) is blocked, and dephosphorylation of cytoplasmic nuclear factor in activated T cells (NFAT) is inhibited resulting in lower levels of IL-2 transcription. IL-2 promotes transcription of B lymphocyte-induced maturation protein-1 (Blimp-1), a co-repressor of B cell lymphoma 6 (Bcl-6). In the absence of IL-2, lower transcription of Blimp-1 leads to increased expression of BCL6 and thus may enhance Tfh cell numbers. Basiliximab may promote the same effect of enhancing Tfh cell numbers *via* blocking the IL-2R.

This limited basis of evidence highlights that there is no clear understanding about the mechanisms by which immunosuppressants affect the development of Tfh cells. CNIs suppress IL-2 production through inhibiting the dephosphorylation of nuclear factor in activated T cells (NFAT), which is the key transcription factor for IL-2 and its receptor (Figure 2) (62). In a mouse model with acute viral infection, Ray *et al*. (39) demonstrated that IL-2 is able to enhance the expression of the transcriptional repressor Blimp-1 through the STAT5 and PI3K–Akt signaling pathway and promotes inhibition of Bcl-6 expression in activated T cells. It ultimately shifts the immune reaction during T-cell differentiation away from a humoral response (Figure 2A). In theory, CNIs may influence Tfh differentiation through the control of IL-2 expression (Figure 2B). Basiliximab may also control Tfh differentiation in the same manner *via* blockade of the IL-2 receptor (Figure 2B). However, as proved in a mouse model, NFAT activity, which is suppressed by Tac, is also a functional requirement for Tfh differentiation and may induce secretion of IL-21 by Tfh cells (63, 64). Since CNIs suppress NFAT activity and the corresponding expression of IL-2 and most likely also of IL-21, this class of agents may influence the generation of Tfh cells through regulating the balance of IL-2 and IL-21. However, further studies are needed to determine the mode of action of immunosuppressive agents on Tfh cells.

## Tfh-Targeted Immunotherapy

### Coinhibitory Pathways

**Figure 3:**
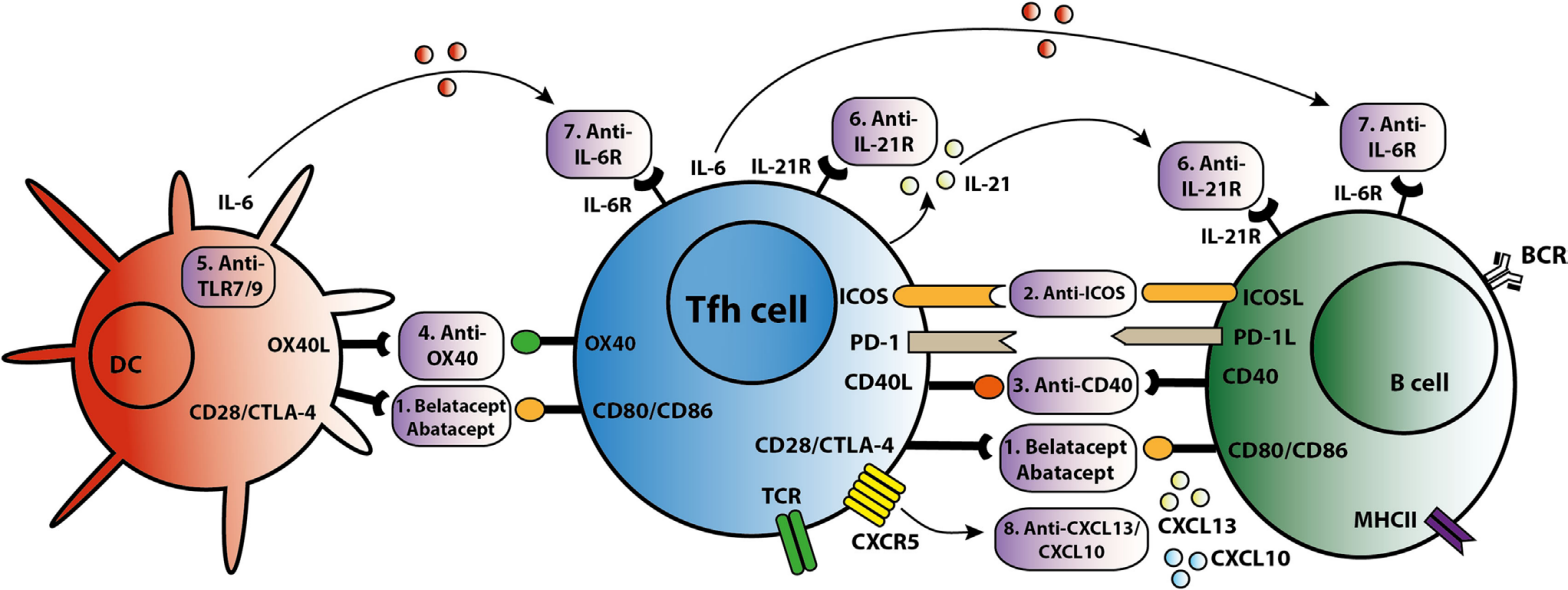
T follicular helper (Tfh)-targeted immunotherapy. Blockage of the Tfh activation and function is established *via* several routes. An overview of these Tfh-targeted immunotherapies is summarized in this figure with (1) belatacept/abatacept, blocking costimulation of CD28/cytotoxic T-lymphocyte-associated protein 4 (CTLA4) and CD80/CD86, (2) anti-inducible T cell costimulatory molecule (ICOS), (3) anti-CD40, (4) anti-OX40, (5) anti toll-like receptor 7 (TLR7) or toll-like receptor 9 (TLR9), (6) anti-IL-21R, (7) anti-IL-6R, and (8) anti-C–X–C chemokine ligand 13 (CXCL13) and anti-CXCL10.

As summarized in Figure 3, different strategies can be employed to target Tfh activation and/or function. CTLA4 could control B cell responses by modulating Tfh-cell activity (30). Abatacept and belatacept are first and second generations of the fusion protein CTLA4-Ig. A study based on a mouse skin graft model by Kim *et al*. showed that abatacept reduced the number of activated CD4^+^PD-1^+^CXCR5^+^ Tfh cells in the spleen, which was associated with suppression of antibodies directed against the skin transplant (65). CTLA4-Ig also inhibited the increase in circulating Tfh cells and B cell-mediated antibody production in a mouse heart transplant model (66). In primary Sjögren’s syndrome patients, abatacept treatment attenuated circulating Tfh-cell numbers and Tfh-cell-dependent B cell hyperactivity (67). In kidney transplant patients, we found that belatacept partially inhibited Tfh-cell activation; the remaining activated Tfh cells were able to provide B cell help. Belatacept is less potent *in vitro* than Tac in inhibiting Tfh-cell-dependent plasmablast formation (60). Based on these preliminary data, it seems that human circulating Tfh cells after transplantation are less susceptible to costimulation blockade than mouse Tfh cells. However, proof that lower susceptibility in humans leads to more extensive antibody-mediated anti-donor responses has not been established. New immune monitoring trials are needed to confirm the immunosuppressive effects of belatacept on human Tfh cells.

### Costimulatory Pathways

T follicular helper cells control the quality of long-lived humoral immunity through the ICOS/ICOSL signaling pathway (68). ICOS is able to directly promote Tfh-cell recruitment toward the GC and acts as a linker between T and B cells, supporting positive selection for high-affinity bone-marrow plasma cells (68, 69). Based on these studies, targeting the ICOS/ICOSL signaling pathway might offer new opportunities to prevent production of DSA and to treat transplant patients with *de novo* DSA. A glyco-engineered antibody that depletes ICOS resulted in a significant reduction in anti-nucleosomal autoantibodies in an SLE lupus-prone mouse model (70). Sato *et al*. demonstrated that ICOS expression was upregulated on T cells in a canine hematopoietic cell transplantation model of graft rejection or chronic graft-versus-host disease. In this study, immunosuppressive effects were observed in mixed leukocyte reactions where anti-ICOS was combined with suboptimal concentrations of CLTA4-Ig or cyclosporine (71). Recently, another study showed that anti-ICOSL antibody did not impact the early Tfh differentiation in a mouse model with *Plasmodium chabaudi* AS infection, but ICOS is necessary for maintenance of a sustained high-affinity, protective Ab response (72). Targeting the ICOS pathway with biologicals is a promising new direction to control the function of Tfh cells and subsequently B cells. However, it is clear that more knowledge is warranted to better understand the reported discrepancies in above described *in vitro* and *in vivo* models. Therefore, the clinical use of anti-ICOS therapy is still in development.

The CD40L/CD40 signaling pathway is also important in the interaction between Tfh and B cells. There are several known anti-CD40 agents. One of these, 2C10R4, is currently being investigated in clinical trials. Kim *et al*. demonstrated in a rhesus macaques kidney transplant model that 2C10R4 prevented AMR *via* affecting Tfh cells and IL-21 production in GCs and reducing production of early *de novo* DSA (73). In this study, belatacept was as effective as 2C10R4 in regulating Tfh cells and preventing acute rejection. More proof that blockade of the CD40–CD40L pathway is effective in inhibiting antibody production comes from studies analyzing the effect of CFZ533, an Fc-silencing and non-B cell depleting anti-CD40 monoclonal antibody. Non-human primates treated with this agent had prolonged kidney allograft survival rates and better kidney function parameters than the untreated control group. Treatment with CFZ533 prevented the production of alloantibodies in these animals (74). Hence, next to CTLA4-Ig, anti-CD40 agents represent a promising option for costimulatory blockade to inhibit both Tfh and B cells. An alternative strategy is the combined use of Tac and new costimulatory blockers to inhibit allograft humoral immunity (73). However, the risk of over-immunosuppression should be considered.

The tumor necrosis factor receptor OX40, activated by its cognate ligand OX40L, functions as a T-cell costimulatory molecule. OX40L is expressed on DCs and myeloid APCs, but not on B cells. CD8α^−^ DCs, known for their MHC class II presentation, are localized at the inter-follicular zone and play a pivotal role in the induction of antigen-specific Tfh cells by upregulating the expression of ICOSL and OX40L (75). The frequency of circulating OX40L-expressing myeloid APCs shows a positive correlation with disease activity and the frequency of ICOS^+^ blood Tfh cells in SLE. This may result from the capacity of OX40 signals to stimulate naïve and memory CD4^+^ T cells to express multiple Tfh cell molecules. Therefore, OX40 signals are adequate to induce these T cells to become functional B cell helpers (76). Blocking the OX40/OX40L pathway resulted in prolonged allograft survival in cardiac and skin transplantation mouse models (77, 78). However, the optimal timing for OX40 blockade is unclear and there are still potential problems with use of OX40/OX40L therapy for posttransplant autoimmunity (79). In addition, OX40L expression on myeloid APCs can be induced by immune complexes containing RNA *via* toll-like receptor 7 (TLR7) activation (76). Toll-like receptor 9 (TLR9) signaling in DCs led to higher numbers of Tfh and GC B cells, and accelerated production of broad-affinity anti-hapten IgG (80). Hence, TLR7/TLR9 might also offer a potential route to prevent antigen presentation of APC to Tfh cells.

### Cytokine-Dependent Pathways

IL-21 acts as the dominate cytokine within the GC and is a promising target to inhibit Tfh function. Treatment of SLE lupus-prone mice with an anti-IL-21 blocking antibody reduced titers of autoantibodies, delayed progression of glomerulonephritis and diminished renal-infiltrating Tfh and Th1 cells, whilst improving overall survival (81). In an allogeneic setting, we recently demonstrated through *ex vivo* experimentation that IL-21 produced by donor antigen-activated Tfh cells could simulate humoral immunity, while an IL-21 receptor antagonist (αIL-21R) could inhibit B cell differentiation and decrease the proportion of plasmablasts and production of IgM and IgG2 (26). Moreover, IL-21 has been shown capable of overcoming Tfr cell-mediated suppression and inhibiting Tfr cells, thus skewing the ratio of Tfh to Tfr cells (40, 82). Since IL-21 is more specific to the interaction of GC–Tfh cells than other cytokines and molecules, there is an urgent need to identify the efficiency and safety of IL-21 inhibition or IL-21R blockade in the suppression of humoral immunity. Both IL-21 receptor antagonists and IL-21 blocking antibodies have the potential to modulate the Tfh-mediated immune response.

IL-6 is a cytokine that offers another attractive option for targeting humoral immunity (23). Specific targeting of IL-6 receptor by tocilizumab in patients with rheumatoid arthritis led to a significant reduction in the number of circulating Tfh cells and in IL-21 production by CD4^+^ T cells (23). In transplantation, anti-IL-6 receptor treatment decreased the number of IL21^+^CD4^+^ and CD4^+^CXCR5^+^ Tfh cells in the spleens of allosensitized mice, as well as suppressing antibody recall responses and DSA levels (83, 84). A first study in kidney transplant patients proved that targeting the IL-6/IL-6R in highly sensitized patients could be a safe and new alternative in addition to currently used plasmapheresis with low-dose intravenous immunoglobulin (85). Given the interplay between IL-6 and IL-21, the combined blockade IL-6 and IL-21 might be a better option to inhibit the differentiation, expansion and function of Tfh cells at all stages.

Transforming growth factor-β appears to have multiple roles in transplantation. It promotes Tfh-cell differentiation (22), while it prevents Tfh-cell accumulation, self-reactive B cell activation and autoantibody production in GCs (86). TGF-β is also a vital fibrosis cytokine (87). A clear understanding of the effect of TGF-β blockade on the entire humoral response and allograft fibrosis is required, however, before any therapeutic application.

### Tfh Migration-Dependent Pathways

Targeting chemokine receptors or ligands to prevent Tfh migration offers another possible route for intervention. C–X–C chemokine ligand 13 (CXCL13) is produced in abundance by follicular stromal cells within B cell follicles. Expression of CXCL13, the receptor for CXCR5, on B and Tfh cells is necessary for their migration to the center of follicles to establish GCs (88). In addition, circulating Tfh cells respond to CXCL13 and can move back to the SLO GC (9). Although CXCL13 acts locally, it can also be detected in plasma and its level is associated with the extent of immune activity (89). Patients with active chronic graft-versus-host disease have a lower frequency of circulating Tfh cells but higher CXCL13 plasma levels, suggesting increased homing of Tfh cells to SLOs (90). Circulating Tfh cells are also able to migrate into allografts *via* other chemokines such as CXCL10, and take part in the formation of tertiary lymphoid organs (9), which have been found in kidney biopsies after rejection (51). Anti-CXCL10 has been used to treat rheumatoid arthritis and ulcerative colitis in phase II clinical trials (91, 92). Depletion of B cells by rituximab has no effect on tertiary lymphoid organs in chronic allograft dysfunction (93). Therefore, targeting CXCL13 or CXCL10 to inhibit the homing and trafficking of Tfh cells might be a more efficient strategy than B cell depletion to prevent the accumulation of Tfh in SLOs and formation of tertiary lymphoid organs in the allograft.

### Immune-Enhancing Pathways

Programmed death 1, which is highly expressed on activated GC–Tfh cells, plays an important role in Tfh cell differentiation and formation of GCs (50). In theory, anti-PD-1 could control Tfh cell activation. However, contradicting results are presented. In a PD-1-deficient mouse model, Kawamoto *et al*. found increased production of Tfh cells with altered phenotypes and dysregulation of GCs, eventually resulting in reduced antigen affinity of IgA (94). Good-Jacobson *et al*. demonstrated that there were less plasma cells, GCs, and Tfh cells in the absence of PD-1; however, the remaining plasma cells were of a higher affinity (95). Others have also observed expansion of Tfh cells and enhanced humoral responses by blocking the PD-1 pathway (96, 97). In general, and thus also in organ transplantation patients, blockade of the PD-1 pathway boosts the immune response. These agents are approved for the treatment of solid tumors (98). A recent case report showed that a kidney transplant recipient given anti-PD-1 treatment for metastatic cutaneous squamous cell carcinoma had an acute cell-mediated allograft rejection two months after starting anti-PD-1, indirectly suggesting a role for PD-1 in regulation of transplant-related immune responses (99). There is no direct evidence that PD-1 agonists or antagonists would benefit transplant patients. The current observations might make PD-1 an unrealistic and ineffective target for achieving allograft humoral immunity.

## Tfh Cells as a Biomarker in Pharmacodynamics

T follicular helper cells may have a role in pharmacodynamic monitoring of immunosuppression, as a marker for the extent to which the antibody response is inhibited. Circulating Tfh cells could be the counterpart of GC–Tfh cells as they share functional properties with Tfh cells residing in SLOs (16). However, whether these circulating Tfh cells are formed in the circulation, or have left the GC after exposure to an antigen and reside as memory Tfh cells in the blood is unknown. He *et al*. demonstrated that the circulating CCR7^lo^PD-1^hi^ Tfh subset correlated with clinical indices in autoimmune diseases and suggested it might serve as a biomarker for pathogenic antibody responses in that setting (100). Bentebibel *et al*. found that the presence of ICOS^+^CXCR3^+^CXCR5^+^CD4^+^ T cells correlated with influenza vaccination antibody responses (101). Several papers have demonstrated that production of the neutralizing HIV antibody is dependent on Tfh cells, which could be a predictive biomarker for vaccine response (19, 102–104). Chenouard *et al*. suggested that a lack of Tfh cells, as seen in kidney transplant patients with operational tolerance, may induce a pro-tolerogenic environment with reduced risk of developing *de novo* DSA (105).

Individualized therapy after transplantation based on pharmacodynamic monitoring is the direction of immunosuppressant development. DSA, the traditional biomarker for humoral immunity, may not be the most sensitive indicator of the immune response after transplantation. As the launching cells for plasmablasts and memory B cells, the frequency of donor-specific Tfh cells might also be a sensitive tool to identify patients at risk for developing a DSA mediated immune response. These donor-specific Tfh cells can be quantified by donor-specific IL-21 enzyme-linked immunospot assay. Perhaps serum IL-21 measured by enzyme linked immunosorbent assay could also be a good alternative for pharmacodynamic monitoring. Of note, CXCL13 has also been suggested as a plasma biomarker for GC activity (106). Dose adjustments and switching immunosuppressive therapies based on monitoring of donor-specific IL-21^+^ Tfh numbers in combination with serum CXCL13 might be of benefit in alloimmune-risk stratification and for individualized therapy in transplantation.

## Perspective and Future Directions

Graft failure in immunosuppressed patients is often a combination of mixed cellular and AMR. Current immunosuppression seems to have limited capacity to control the allograft loss in recipients with high risk of rejection. Targeting the Tfh cell during maintenance therapy has the potential to prevent mixed clinical rejection early in the activation cascade, due to its pivotal role in conjugating the interaction of T and B cells. Suppression of both GC–Tfh cell formation and Tfh-cell migration *via* two different agents—for example, by blocking IL-21 and CXCL13—probably provides more robust control of humoral immunity. In addition, treatment strategies for patients undergoing solid organ transplantation should be defined according to the individual’s immune activity (*e.g*., number of circulating donor-specific IL-21^+^ T cells) and risk of rejection. Monitoring donor-specific Tfh and memory B cells to guide the implementation of immunosuppression would help to tailor therapy appropriately. It would be of high interest to develop tools to easily investigate the specificity of these Tfh cells. In addition, immunosuppressive agents such as mTOR inhibitors with known Treg generating effects might also beneficially contribute to the generation of antigen-specific Tfr cells in transplant patients (107). This recently described T cell population controlling B cell immune responses is of high interest to intervene in the humoral alloimmune response leading to improved graft survival in patients after organ transplantation (27).

Although many agents can influence Tfh cell development and function, only a few are likely to be suitable for clinical investigation. Successful immunosuppressive drug development has a lifecycle that includes the discovery of a target molecule, research into the mechanisms involved, animal experimentation, and validation in clinical trials. Clinical assessment requires comparison of the new drug versus current therapies and monitoring of adverse effects. Comparing the effects of new Tfh-targeting therapeutics against the conventional immunosuppressants used in transplantation, particularly in terms of Tfh-dependent humoral immune activity, would be of considerable interest.

## Author Contributions

LY and KL researched the literature, evaluated evidence, and wrote the manuscript. RH, LL, YS, LW, and CB evaluated evidence and edited the manuscript.

## Conflict of Interest Statement

The authors declare that the research was conducted in the absence of any commercial or financial relationships that could be construed as a potential conflict of interest.

## Abbreviations

AMR, antibody-mediated rejection; APC, antigen-presenting cell; Bcl-6, B cell lymphoma 6; BCR, B cell receptor; Blimp-1, B lymphocyte-induced maturation protein-1; BTK, Bruton’s tyrosine kinase; CNI, calcineurin inhibitor; CTLA4, cytotoxic T-lymphocyte-associated protein 4; CXCL13, C–X–C chemokine ligand 13; CXCR5, C–X–C chemokine receptor 5; DC, dendritic cell; DZ, dark zone; DSA, donor-specific antibodies; GC, germinal center; GOF, gain of function; ICOS, inducible T cell costimulatory molecule; LEF-1, lymphoid enhancer binding factor 1; LOF, loss of function; LRBA, LPS-responsive beige-like anchor; LZ, light zone; MMF, mycophenolate mofetil; NFAT, nuclear factor in activated T cells; PD-1, programmed death 1; PID, primary immunodeficiency; SHM, somatic hypermutation; SLO, secondary lymphoid organs; STAT, signal transducer and activator of transcription; TCF-1, T cell factor 1; Tfh cell, T follicular helper cell; Tfr, T follicular regulatory; TGF-β, transforming growth factor-β; TLR7, toll-like receptor 7; TLR9, toll-like receptor 9; Treg, regulatory T.
